# Supplementary material for: Multi‐dataset identification of innovative feature genes and molecular mechanisms in keratoconus
Source: J Cell Mol Med. 2024 Sep 19;28(18):e70079. doi: 10.1111/jcmm.70079 (PMC11412914; doi:10.1111/jcmm.70079)
Supplement: Supplementary file 2 — Material S2. [file JCMM-28-e70079-s002.zip › 7.GSEA analysis/5.RNF217-AS1/KEGG_LYSOSOME.html]

Details for gene set KEGG\_LYSOSOME[GSEA]

|  || Dataset | RNF217-AS1.group.cls#h\_versus\_l |
| Phenotype | group.cls#h\_versus\_l |
| Upregulated in class | l |
| GeneSet | KEGG\_LYSOSOME |
| Enrichment Score (ES) | -0.29004708 |
| Normalized Enrichment Score (NES) | -1.5075566 |
| Nominal p-value | 0.093023255 |
| FDR q-value | 0.3045308 |
| FWER p-Value | 0.751 |
Table: GSEA Results Summary

  

Fig 1: Enrichment plot: KEGG\_LYSOSOME      
 Profile of the Running ES Score & Positions of GeneSet Members on the Rank Ordered List

  

| SYMBOL | TITLE | RANK IN GENE LIST | RANK METRIC SCORE | RUNNING ES | CORE ENRICHMENT || 1 | CLTC | na | 2152 | 0.337 | -0.0227 | No |
| 2 | ATP6V0D2 | na | 2490 | 0.317 | 0.0047 | No |
| 3 | ACP5 | na | 5694 | 0.193 | -0.0653 | No |
| 4 | AP1S1 | na | 6712 | 0.161 | -0.0758 | No |
| 5 | CTSW | na | 6821 | 0.158 | -0.0604 | No |
| 6 | DNASE2B | na | 6970 | 0.154 | -0.0466 | No |
| 7 | ATP6V0D1 | na | 9204 | 0.101 | -0.0993 | No |
| 8 | CTSG | na | 9428 | 0.097 | -0.0944 | No |
| 9 | CTNS | na | 9818 | 0.089 | -0.0952 | No |
| 10 | ARSB | na | 11447 | 0.064 | -0.1348 | No |
| 11 | CTSL | na | 11878 | 0.057 | -0.1405 | No |
| 12 | GGA1 | na | 12289 | 0.052 | -0.1463 | No |
| 13 | AP1G1 | na | 12799 | 0.045 | -0.1558 | No |
| 14 | MFSD8 | na | 13333 | 0.038 | -0.1667 | No |
| 15 | AP3M1 | na | 13805 | 0.033 | -0.1765 | No |
| 16 | IGF2R | na | 13821 | 0.032 | -0.1731 | No |
| 17 | NPC1 | na | 14253 | 0.028 | -0.1824 | No |
| 18 | GM2A | na | 14277 | 0.027 | -0.1798 | No |
| 19 | AP4M1 | na | 15102 | 0.018 | -0.2015 | No |
| 20 | CLN3 | na | 15109 | 0.018 | -0.1996 | No |
| 21 | IDUA | na | 15130 | 0.018 | -0.1981 | No |
| 22 | CLTA | na | 15454 | 0.014 | -0.2057 | No |
| 23 | CTSB | na | 15528 | 0.014 | -0.2062 | No |
| 24 | ATP6V0B | na | 15585 | 0.013 | -0.2063 | No |
| 25 | GAA | na | 15679 | 0.012 | -0.2076 | No |
| 26 | TPP1 | na | 15779 | 0.011 | -0.2092 | No |
| 27 | MCOLN1 | na | 16182 | 0.007 | -0.2200 | No |
| 28 | ENTPD4 | na | 16251 | 0.006 | -0.2212 | No |
| 29 | ABCA2 | na | 16445 | 0.004 | -0.2263 | No |
| 30 | GGA2 | na | 16498 | 0.004 | -0.2273 | No |
| 31 | PSAP | na | 16568 | 0.003 | -0.2290 | No |
| 32 | CD164 | na | 16590 | 0.003 | -0.2293 | No |
| 33 | GALNS | na | 16817 | 0.000 | -0.2357 | No |
| 34 | ARSG | na | 16975 | -0.001 | -0.2401 | No |
| 35 | AP3D1 | na | 17050 | -0.002 | -0.2420 | No |
| 36 | GALC | na | 17091 | -0.003 | -0.2429 | No |
| 37 | SLC11A1 | na | 17203 | -0.004 | -0.2457 | No |
| 38 | AP1S3 | na | 17232 | -0.004 | -0.2460 | No |
| 39 | CLN5 | na | 17400 | -0.005 | -0.2502 | No |
| 40 | AP3S1 | na | 17594 | -0.007 | -0.2549 | No |
| 41 | GNPTAB | na | 17687 | -0.008 | -0.2567 | No |
| 42 | AP4E1 | na | 17740 | -0.008 | -0.2572 | No |
| 43 | GGA3 | na | 17912 | -0.010 | -0.2610 | No |
| 44 | HEXA | na | 18191 | -0.013 | -0.2675 | No |
| 45 | LAPTM4B | na | 18662 | -0.017 | -0.2791 | No |
| 46 | ATP6V0C | na | 18707 | -0.017 | -0.2783 | No |
| 47 | AGA | na | 18821 | -0.019 | -0.2794 | No |
| 48 | AP4S1 | na | 18979 | -0.020 | -0.2816 | No |
| 49 | CD63 | na | 19044 | -0.020 | -0.2811 | No |
| 50 | LAMP1 | na | 19048 | -0.020 | -0.2788 | No |
| 51 | NAPSA | na | 19088 | -0.021 | -0.2775 | No |
| 52 | LAMP2 | na | 19102 | -0.021 | -0.2754 | No |
| 53 | NEU1 | na | 19319 | -0.023 | -0.2789 | No |
| 54 | HGSNAT | na | 19338 | -0.024 | -0.2767 | No |
| 55 | AP1M2 | na | 19492 | -0.025 | -0.2781 | No |
| 56 | SGSH | na | 19558 | -0.026 | -0.2770 | No |
| 57 | SUMF1 | na | 19664 | -0.027 | -0.2769 | No |
| 58 | GUSB | na | 19854 | -0.029 | -0.2790 | No |
| 59 | CTSV | na | 19926 | -0.029 | -0.2777 | No |
| 60 | AP3S2 | na | 20356 | -0.033 | -0.2861 | Yes |
| 61 | ATP6AP1 | na | 20488 | -0.035 | -0.2858 | Yes |
| 62 | PSAPL1 | na | 20490 | -0.035 | -0.2818 | Yes |
| 63 | ARSA | na | 20565 | -0.036 | -0.2798 | Yes |
| 64 | CLTB | na | 20606 | -0.036 | -0.2767 | Yes |
| 65 | LAPTM4A | na | 20624 | -0.036 | -0.2730 | Yes |
| 66 | GNPTG | na | 20786 | -0.038 | -0.2732 | Yes |
| 67 | GNS | na | 21215 | -0.042 | -0.2806 | Yes |
| 68 | CTSA | na | 21264 | -0.043 | -0.2770 | Yes |
| 69 | SORT1 | na | 21315 | -0.043 | -0.2734 | Yes |
| 70 | IDS | na | 21328 | -0.043 | -0.2686 | Yes |
| 71 | M6PR | na | 21430 | -0.044 | -0.2663 | Yes |
| 72 | ATP6V1H | na | 21597 | -0.046 | -0.2657 | Yes |
| 73 | ATP6V0A1 | na | 21733 | -0.048 | -0.2640 | Yes |
| 74 | CTSC | na | 22020 | -0.051 | -0.2663 | Yes |
| 75 | NPC2 | na | 22051 | -0.051 | -0.2612 | Yes |
| 76 | CTSD | na | 22142 | -0.052 | -0.2577 | Yes |
| 77 | PPT2 | na | 22180 | -0.053 | -0.2526 | Yes |
| 78 | AP1B1 | na | 22198 | -0.053 | -0.2469 | Yes |
| 79 | HEXB | na | 22880 | -0.060 | -0.2595 | Yes |
| 80 | CTSH | na | 23058 | -0.063 | -0.2573 | Yes |
| 81 | PLA2G15 | na | 23074 | -0.063 | -0.2503 | Yes |
| 82 | HYAL1 | na | 23159 | -0.064 | -0.2453 | Yes |
| 83 | AP3B1 | na | 23179 | -0.064 | -0.2384 | Yes |
| 84 | GLA | na | 23202 | -0.064 | -0.2315 | Yes |
| 85 | LIPA | na | 23664 | -0.070 | -0.2366 | Yes |
| 86 | PPT1 | na | 23956 | -0.074 | -0.2363 | Yes |
| 87 | MANBA | na | 23980 | -0.075 | -0.2282 | Yes |
| 88 | ASAH1 | na | 23989 | -0.075 | -0.2197 | Yes |
| 89 | NAGLU | na | 24000 | -0.075 | -0.2111 | Yes |
| 90 | CTSK | na | 24029 | -0.076 | -0.2031 | Yes |
| 91 | MAN2B1 | na | 24293 | -0.080 | -0.2014 | Yes |
| 92 | FUCA1 | na | 24315 | -0.080 | -0.1926 | Yes |
| 93 | SCARB2 | na | 24341 | -0.080 | -0.1839 | Yes |
| 94 | SLC17A5 | na | 24684 | -0.086 | -0.1838 | Yes |
| 95 | NAGPA | na | 24922 | -0.089 | -0.1802 | Yes |
| 96 | ACP2 | na | 25046 | -0.091 | -0.1731 | Yes |
| 97 | SLC11A2 | na | 25282 | -0.095 | -0.1688 | Yes |
| 98 | NAGA | na | 25389 | -0.097 | -0.1605 | Yes |
| 99 | AP3M2 | na | 25733 | -0.102 | -0.1584 | Yes |
| 100 | AP1M1 | na | 25781 | -0.103 | -0.1477 | Yes |
| 101 | AP1S2 | na | 25797 | -0.103 | -0.1361 | Yes |
| 102 | DNASE2 | na | 25914 | -0.105 | -0.1271 | Yes |
| 103 | CLTCL1 | na | 26347 | -0.113 | -0.1263 | Yes |
| 104 | CTSO | na | 26357 | -0.114 | -0.1132 | Yes |
| 105 | GLB1 | na | 26907 | -0.124 | -0.1146 | Yes |
| 106 | CTSF | na | 27501 | -0.137 | -0.1158 | Yes |
| 107 | SMPD1 | na | 28656 | -0.167 | -0.1296 | Yes |
| 108 | AP4B1 | na | 28808 | -0.171 | -0.1140 | Yes |
| 109 | LGMN | na | 28885 | -0.173 | -0.0959 | Yes |
| 110 | CTSZ | na | 29702 | -0.197 | -0.0964 | Yes |
| 111 | ATP6V0A4 | na | 30469 | -0.223 | -0.0925 | Yes |
| 112 | CTSS | na | 30929 | -0.239 | -0.0778 | Yes |
| 113 | CTSE | na | 31449 | -0.261 | -0.0622 | Yes |
| 114 | ABCB9 | na | 32246 | -0.306 | -0.0494 | Yes |
| 115 | LAPTM5 | na | 32583 | -0.330 | -0.0205 | Yes |
| 116 | LAMP3 | na | 32853 | -0.349 | 0.0126 | Yes |
| 117 | AP3B2 | na | 32926 | -0.355 | 0.0521 | Yes |
Table: GSEA details [plain text format]

  

Fig 2: KEGG\_LYSOSOME      
 Blue-Pink O' Gram in the Space of the Analyzed GeneSet

  

Fig 3: KEGG\_LYSOSOME: Random ES distribution      
 Gene set null distribution of ES for **KEGG\_LYSOSOME**
